# Hop biomass waste against fire blight and black rot: an eco-friendly approach to crop protection

**DOI:** 10.3389/fmicb.2025.1665767

**Published:** 2025-11-07

**Authors:** Riccardo Fontana, Leandra Leto, Maria Grazia Ortore, Valeria Guarrasi, Walter Pula, Elisabetta Esposito, Francesco Bigi, Benedetta Chiancone, Peggy Marconi

**Affiliations:** 1 Department of Chemical, Pharmaceutical and Agricultural Sciences, University of Ferrara, Ferrara, Italy; 2 Department of Food and Drug Sciences, University of Parma, Parma, Italy; 3 Department of Life and Environmental Sciences, Marche Polytechnic University, Ancona, Italy; 4 Institute of Biophysics, National Research Council (CNR), Palermo, Italy; 5 Packtin srl, Reggio Emilia, Italy; 6 Department of Environmental and Prevention Sciences, University of Ferrara, Ferrara, Italy; 7 LTTA Laboratory for Advanced Therapies, Technopole of Ferrara, Ferrara, Italy

**Keywords:** *Erwinia amylovora*, *Humulus lupulus*, biomass waste, phytopathogens, *Xanthomonas campestris*, virulence factors

## Abstract

Hop is a perennial, deciduous climbing plant best known for its use in beer production. Recently, in line with the Green Strategy of Agenda 2030 and increasing market demand, research and industry interest in natural products has grown. In this context, hop biomass waste (HW), such as leaves, stems and non-standardized cones discarded after cone harvesting, has been shown to contain a wealth of bioactive compounds. These compounds offer promising applications in several productive sectors, including the pesticide industry. This study aims to evaluate the effect of extracts obtained from powdered HW (HWP) on two quarantine phytopathogenic bacteria: *Erwinia amylovora* (EA), the causal agent of fire blight in Rosaceae, and *Xanthomonas campestris* pv. *campestris* (Xcc), the causal agent of black rot in crucifers. Both diseases are highly destructive and economically impactful, necessitating the search for effective and eco-friendly alternatives. Various extracts were prepared, including two hydroalcoholic ones (ethanol/water 80:20 and 70:30), one ethanolic extract, and one methanolic extract. The potential mechanisms of action were investigated, with a focus on bacterial membrane permeability, biofilm formation and removal, and antioxidant activity, assessed using the Folin–Ciocalteu test. The *in vitro* analyses demonstrated that HWP extracts exhibit significant antibacterial activity against both EA and Xcc by altering bacterial membrane permeability, inhibiting biofilm formation, and promoting biofilm removal. The Folin–Ciocalteu test confirmed the presence of polyphenols, suggesting antioxidant activity. In planta tests provided realistic insights into the interaction between plants, bacteria, and extracts, showing promising results for the use of HWP extracts in controlling these phytopathogens. The study highlights the high efficacy, eco-friendliness, and sustainability of low-cost HWP extracts as a potential strategy for controlling fire blight and black rot. HWP extracts could serve as an alternative to traditional synthetic pesticides and antibiotics, promoting more sustainable and environmentally friendly agricultural practices, in line with Sustainable Development Goal 2 of the 2030 Agenda.

## 1 Introduction

Plant diseases caused by phytopathogens represent a growing threat to global agriculture, leading to substantial economic losses and impacting food security. Among these pathogens, bacteria such as *Xanthomonas campestris* pv. *campestris* (Xcc) and *Erwinia amylovora* (EA) are especially known for their virulence and the severe diseases they induce in crops (Vicente and Holub, 2013; Vanneste, 2001).

*Xanthomonas campestris* pv. *campestris* (Xcc) is the causative agent of black rot in cruciferous vegetables, including cabbage, broccoli, and cauliflower (Liu et al., 2022). This pathogen uses various virulence factors to infect host plants. Key factors in this process include the type III secretion system (T3SS) and its associated effector proteins, which enable the injection of bacterial proteins directly into plant cells, subverting host defenses and promoting infection (Zhou et al., 2020). Additionally, Xcc produces extracellular polysaccharides (EPS), such as xanthan gum, which contribute to biofilm formation and protect the bacteria from plant immune responses. Lipopolysaccharides (LPS) in the bacterial outer membrane also play a crucial role in biofilm development, evading host detection and enhancing virulence (Vorhölter et al., 2001). The impact of these virulence factors is profound, leading to the characteristic V-shaped lesions on leaves, systemic infection, and significant yield losses in affected crops.

Similarly, *Erwinia amylovora* (EA), the pathogen responsible for fire blight in members of the *Rosaceae* family, such as apple and pear trees, utilizes a sophisticated arsenal of virulence factors (Vanneste, 2022). The T3SS in *EA* is essential for its pathogenicity, delivering effector proteins that manipulate host cellular processes to promote bacterial colonization. Another critical virulence factor is amylovoran, an exopolysaccharide that facilitates biofilm formation and protects the bacteria from environmental stresses and host immune responses. Additionally, *EA* employs quorum-sensing mechanisms to coordinate the expression of virulence genes in response to population density, enhancing its ability to cause disease. The rapid spread and severe symptoms of fire blight, including shoot blight and fruit mummification, highlight the devastating impact of these virulence factors on orchard productivity (Piqué et al., 2015).

In the pursuit of sustainable disease management, the use of plant-derived compounds has garnered significant interest (Astray et al., 2020). This growing interest has the development of sustainable production models and an increasing emphasis on food safety, driving integrated pest management (IPM) efforts and the use of plant extracts as “green agrochemical products.” By evaluating the richness of bioactive compounds in these extracts, IPM strategies can improve pest control efficacy while promoting ecological balance, ultimately contributing to resilient agricultural systems consistent with sustainability goals.

*Humulus lupulus* L., commonly known as hops, is a plant renowned for its antimicrobial properties. Hop extracts, mainly derived from the cones of *Humulus lupulus* L., but not exclusively, contain a wide range of bioactive compounds that contribute to their antimicrobial effects (Astray et al., 2020; Zanoli and Zavatti, 2008). The key components include alpha acids, such as humulone, cohumulone, and adhumulone, which are the primary bitter compounds in hops and are known for their antimicrobial activity, especially against Gram-positive bacteria. Beta acids, including lupulone, colupulone, and adlupulone, also have antimicrobial properties and, while less bitter than alpha acids, significantly enhance the overall antimicrobial efficacy of hop extracts. Besides these acids, hop extracts are rich in essential oils like myrcene, humulene, caryophyllene, and farnesene. These oils not only provide the characteristic aroma of hops but also exhibit antimicrobial and antioxidant properties. Polyphenols, such as flavonoids and phenolic acids like quercetin and xanthohumol, are another crucial component. These compounds are known for their antioxidant properties, which help neutralize reactive oxygen species and defend cells from oxidative damage. Terpenes and terpenoids, including compounds like limonene and linalool, contribute to the aromatic profile of hops but also have demonstrated antimicrobial and anti-inflammatory properties (Bocquet et al., 2019; Di Sotto et al., 2018). Additionally, hop extracts contain minor components such as hard resins, waxes, chlorophylls, and inorganic salts, which can also contribute to their overall bioactivity.

The antimicrobial properties of hop extracts are particularly noteworthy: *α*- and *β*-acids disrupt bacterial cell membranes, leading to cell lysis and death; essential oils and polyphenols further enhance this effect by interfering with bacterial metabolism and protecting against oxidative stress.

Recent studies have focused not only on extracts from hop cones but also on the characterization and utilization of bioactive compounds in hop leaves. Traditionally, after harvesting the cones, large amounts of hop biomass, including leaves and stems, are treated as waste and composted; however, this biomass is rich in phenolic compounds such as flavonols, prenylflavonoids and proanthocyanidins (Chiancone et al., 2023; Leto et al., 2024; Sabbatini et al., 2024; Macchioni et al., 2021). These compounds possess antioxidant, antimicrobial, and estrogenic effects (Santarelli et al., 2021) and are produced by the plant as part of its natural defense mechanisms against biotic and abiotic stress factors (Kunej et al., 2020). These properties make hop biomass extracts a promising natural alternative for managing plant diseases and reducing reliance on synthetic chemicals (Karabín et al., 2016). Incorporating hop extracts into disease management strategies not only leverages their antimicrobial properties but also aligns with sustainable agricultural practices, reducing the environmental impact of chemical pesticides and promoting healthier crop production systems. Understanding the virulence mechanisms of phytopathogens like Xcc and EA, along with exploring the antimicrobial potential of plant extracts such as those from *Humulus lupulus* agricultural waste, is crucial for developing effective and sustainable approaches to plant disease management and favoring a circular economy.

In this study, powdered hop biomass waste was used to produce extracts that could serve as innovative solutions for protecting crops and vegetables from bacterial diseases.

## 2 Materials and methods

### 2.1 Plant material and extracts preparation

The *Cascade* hop plants were grown at Azienda Agricola Ludovico Lucchi in Campogalliano (MO), Emilia-Romagna, Italy (44°42′19.9″N, 10°50′26.1″E). This American aroma variety, introduced in 1972 by Oregon State University and the USDA (Padgitt-Cobb et al., 2021), originates from a breeding program in which Fuggle was crossed with a hybrid of Serebrianka and a Fuggle seedling, followed by open pollination (Henning et al., 2018).

Fresh hop biomass waste, including stems, leaves and discarded cones, were dried at 35°C in a low-temperature dryer (model PKT 2022, Packtin srl, Reggio Emilia, Italy) until a relative humidity of 6.0 ± 0.5% was reached. The dried material was then ground with a hammer mill (model Rapide 200Z, Nuova Guseo S.r.l., Villanova sull’Arda, Italy), and sieved through a 1,000 μm fine sieve to obtain a fine powder (HWP), which was stored at room temperature in low-density polyethylene bags.

For the next employment of this matrix, we used ethanol, methanol, which were purchased from Sigma-Aldrich, and distilled water. All chemicals used were of analytical grade (≥99%). The hop extracts subsequently prepared and described in the next paragraphs were tested in a final concentration range of 10 mg/mL to 1 μg/mL, using stock solutions initially prepared at 400 mg/mL in the respective solvents (ethanol, methanol, 80:20, and 70:30 ethanol: water). These stock solutions were then diluted in sterile distilled water prior to application in all biological assays. As a result, although the initial solvent used for extraction and stock preparation had high ethanol content (e.g., 80%), the final solvent concentration in the wells was extremely low, typically <1% v/v, and insufficient to exert any antimicrobial effect on the cultures. This was also confirmed by the solvent-only controls, which showed no measurable inhibition of bacterial growth, thus ensuring that the biological effects observed were due to the active compounds in the hop extracts and not the solvent itself.

#### 2.1.1 Ethanolic extraction

10 g of HWP were mixed with 100 mL of ethanol. The mixture was treated with ultrasound at a power of 87.5 for 30 min at room temperature. Subsequently, it was macerated at 40°C for 10 min under constant rotation, which was repeated three times with fresh ethanol. After each maceration step, the extracts were filtered through a Büchner funnel using Whatman filter paper No. 1. The combined filtrates (300 mL) were completely evaporated under reduced pressure using a rotary evaporator. The residue was weighted, resuspended in ethanol and stored at −20°C until further analysis. For microbiological assays, the extracts, initially solubilized in a minimal volume of ethanol, were subsequently diluted with sterile distilled water to achieve the desired final concentrations, thereby minimizing any solvent-related effects on bacterial growth (Hrnčič et al., 2019; Panzella et al., 2020). The extraction yield was 8.6% (w/w). All solvents were evaporated under reduced pressure using a rotary evaporator. The final dried residues were resuspended in the same solvent used for extraction and stored at −20°C. No specific solvent residue analysis (e.g., GC–MS or ethanol quantification) was performed on the final extracts.

#### 2.1.2 Hydroalcoholic extraction (80:20)

10 g of HWP were mixed with 100 mL of an 80:20 (v/v) ethanol/water solution. The mixture was treated with ultrasound at a power of 87.5 for 30 min at room temperature. Subsequently, it was macerated at 40°C for 10 min under continuous rotation, which was repeated three times with fresh 80:20 ethanol-water solution. After each maceration step, the extracts were filtered through a Büchner funnel using Whatman filter paper No. 1. The combined filtrates were evaporated under reduced pressure using a rotary evaporator. The residue was weighted, resuspended in ethanol and water and stored at −20°C until further analysis. For microbiological assays, the extracts, initially solubilized in a minimal volume of hydroalcoholic solution, were subsequently diluted with sterile distilled water to achieve the desired final concentrations, thereby minimizing any solvent-related effects on bacterial growth. The extraction yield was 10.2% (w/w). All solvents were evaporated under reduced pressure using a rotary evaporator. The final dried residues were resuspended in the same solvent used for extraction and stored at −20°C. No specific solvent residue analysis (e.g., GC–MS or ethanol quantification) was performed on the final extracts.

#### 2.1.3 Hydroalcoholic extraction (70:30)

10 g of HWP were mixed with 100 mL of a 70:30 (v/v) ethanol/water solution. The mixture was treated with ultrasound at a power of 87.5 for 30 min at room temperature. Subsequently, the HWP was macerated at 40°C for 10 min under continuous rotation, which was repeated three times with fresh 70:30 ethanol-water solution. After each maceration step, the extracts were filtered through a Büchner funnel using Whatman filter paper No. 1. The combined filtrates were evaporated under reduced pressure using a rotary evaporator. The residue was weighted, resuspended in ethanol and water and stored at −20°C until further analysis. For microbiological assays, the extracts, initially solubilized in a minimal volume of the hydroalcoholic solution, were subsequently diluted with sterile distilled water to achieve the desired final concentrations, thereby minimizing any solvent-related effects on bacterial growth. The extraction yield was 9.1% (w/w). All solvents were evaporated under reduced pressure using a rotary evaporator. The final dried residues were resuspended in the same solvent used for extraction and stored at −20°C. No specific solvent residue analysis (e.g., GC–MS or ethanol quantification) was performed on the final extracts.

#### 2.1.4 Methanolic extraction

10 g of HWP were mixed with 100 mL of methanol. The mixture was treated with ultrasound at a power of 87.5 for 30 min at room temperature. Subsequently, the hop powder was macerated at 40°C for 10 min under constant rotation, which was repeated three times with fresh methanol. After each maceration step, the extracts were filtered through a Büchner funnel using Whatman filter paper No. 1. The combined filtrates were evaporated under reduced pressure using a rotary evaporator. The residue was weighted, resuspended in methanol and stored at −20°C until further analysis. For microbiological assays, the extracts, initially solubilized in a minimal volume of methanol, were subsequently diluted with sterile distilled water to achieve the desired final concentrations, thereby minimizing any solvent-related effects on bacterial growth. The extraction yield was 7.8% (w/w). All solvents were evaporated under reduced pressure using a rotary evaporator. The final dried residues were resuspended in the same solvent used for extraction and stored at −20°C. No specific solvent residue analysis (e.g., GC–MS or ethanol quantification) was performed on the final extracts.

### 2.2 Chemical analysis

#### 2.2.1 Total polyphenol content

The total phenolic content (TPC) was measured using the Folin–Ciocalteu method based on Martelli et al. (2020). For this analysis, 250 μL of the extract was mixed with 1 mL of a 1:10 (v/v) dilution of Folin–Ciocalteu phenol reagent (Sigma-Aldrich, St. Louis, MO, United States) and 2 mL of a 20% (w/v) aqueous sodium carbonate solution. The mixture was allowed to stand in the dark for 30 min and then the absorbance was measured at 760 nm using a JASCO V-530 spectrophotometer (Easton, MD, United States).

A gallic acid calibration curve with five concentration points (10–100 mg/kg) was used as a standard reference to quantify the polyphenols. Each sample extract was tested in triplicate, with results expressed as milligrams of gallic acid equivalents per gram of sample (mg GAE/g).

#### 2.2.2 Antioxidant activity of extracts

The antioxidant activity of the extracts was evaluated using a DPPH radical scavenging assay based on the method described by Abram et al. (2015) with slight adaptations. For this procedure, 100 μL of each sample, different extracts, or standard solution was added to 2.9 mL of a 0.05 mM ethanolic DPPH solution (Sigma-Aldrich, St. Louis, MO, United States). The solution was incubated in the dark for 30 min, and the absorbance was then recorded at 517 nm using a JASCO V-530 spectrophotometer (Easton, MD, United States). Trolox (6-hydroxy-2,5,7,8-tetramethylchroman-2-carboxylic acid) from Sigma-Aldrich was used to generate a calibration curve for quantification of antioxidant activity, with standard solutions ranging from 0.1 to 1 mM. A control sample consisting in ascorbic acid (ASC) was used. A blank sample containing only 100 μL of the extraction solution was also analyzed under the same conditions.

### 2.3 *In vitro* antimicrobial properties of extracts

#### 2.3.1 Tested microorganisms

The EA and Xcc isolates employed in this study were kindly supplied by the Plant Protection Agency of Emilia-Romagna, whereas the reference strains DSM 30165 and DSM 3586, obtained from the Leibniz Institute DSMZ-German Collection of Microorganisms, were used as controls. Bacterial cultures were maintained on Luria Bertani (LB) agar (30 g/L), R2A agar (specifically for Xcc), or in LB broth (Liofilchem, Roseto degli Abruzzi, Italy), with incubation temperatures consistently maintained between 25 and 28°C. Inoculum concentrations were selected in accordance with established protocols and previously reported data, as referenced in the corresponding sections. For biofilm formation assays, a higher inoculum density of 10^6^ CFU/mL was employed to enhance the robustness and expedite the development of biofilms. A similarly elevated inoculum was utilized in the in-planta trials. Propidium iodide (PI) and cetylpyridinium chloride (CPC) assays were conducted using bacterial suspensions ranging from 10^4^ to 10^7^ CFU/mL to assess membrane permeability and amylovoran production. Following preliminary analyses and in accordance with the literature, an inoculum concentration of 10^5^ CFU/mL was subsequently selected as the standardized condition for downstream applications.

#### 2.3.2 Determination of minimum inhibitory concentration (MIC) and minimum bactericidal concentration (MBC)

The minimum inhibitory concentration (MIC) of the hop water phase (HWP) extracts was determined using the broth microdilution method, following the guidelines established by the European Committee on Antimicrobial Susceptibility Testing (EUCAST) (European Committee for Antimicrobial Susceptibility Testing, 2003). Initially, EA cultures were grown overnight at 28°C in LB broth with constant agitation at 160 rpm in a MaxQ 4,000 incubator (Thermo Scientific Italia, Milan, Italy). Aliquots of the HWP extract stock solutions were subsequently added to LB broth to achieve an initial concentration of 10,000 μg/mL in the first well of each row in sterile 96-well microtiter plates (Promega, Madison, WI, United States). Serial twofold dilutions were then performed in LB medium directly within the microplate, generating a concentration gradient from 10,000 to 5 μg/mL, with a final volume of 200 μL per well. Each well was then inoculated with 10 μL of the overnight bacterial suspension, resulting in a standardized inoculum of 10^4^ CFU/mL. Plates were incubated under static conditions at 25°C for 48 h. Bacterial growth was subsequently assessed by measuring turbidity at OD₆₀₀ using a GloMax® spectrophotometer (Promega, Madison, WI, United States). The same experimental procedure was applied to Xcc. Data represent the mean values from three independent experiments; each conducted in triplicate. Streptomycin and tetracycline, used at their respective MICs reported in the literature, were included as reference antimicrobial agents. Solvent-only treatments were included as negative controls to isolate the effect of hop-derived compounds from the intrinsic antimicrobial activity of the solvents themselves.

#### 2.3.3 Biofilm studies: biofilm formation and removal activity of the extracts/antibiofilm activity

EA and Xcc are recognized for their pronounced capacity to form biofilms, a pivotal process in their pathogenic life cycle. The impact of hop extracts on biofilm development was assessed using the crystal violet microplate assay, following the methodology outlined by Wilson et al. (2017). EA cultures were adjusted to a final concentration of 10^6^ CFU/mL in LB broth; for biofilm inhibition assays, subinhibitory concentrations (subMICs) of hop extracts were incorporated into each well of a 96-well U-bottom microplate. Plates were incubated statically at 25°C for 72 h to allow biofilm formation. Upon completion of incubation, the medium, hop extracts, and planktonic cells were carefully aspirated, and wells were gently rinsed with deionized water to remove residual non-adherent bacteria. For biofilm disruption assays, hop extracts were introduced at this stage onto pre-formed biofilms and incubated for an additional 24 h under the same conditions. Following treatment, a 1% crystal violet solution was added to each well and incubated at room temperature for 30 min to stain the adherent biofilm matrix. Excess stain was removed by successive washes with deionized water. To solubilize the bound dye, 200 μL of 95% ethanol was added per well and incubated for 15 min at room temperature. The contents were then transferred to a clean, sterile microplate, and the absorbance was measured at 570 nm using a microplate reader (Promega, Madison, WI, United States). The same protocol was applied to Xcc. Quantitative analyses were based on three independent biological replicates, each performed in triplicate.

#### 2.3.4 Membrane permeability test

EA bacterial suspensions were cultured in LB broth at 25°C for 24 h. Subsequently, aliquots containing 10^5^ CFU/mL were transferred into individual Eppendorf tubes supplemented with hop extracts at their respective MIC and subinhibitory (½ MIC) concentrations. These suspensions were incubated for 180, 120, 60, and 5 min. Following incubation, samples were centrifuged at 10,000 rpm for 5 min and washed with phosphate-buffered saline (PBS). The resulting pellets were resuspended in a 0.5% solution of propidium iodide (PI) and incubated for 15 min in the dark to prevent photoactivation. Fluorescence intensity was then measured in 96-well black microplates using a fluorescence microplate reader (Promega, Madison, WI, United States) (Crowley et al., 2016; Kirchhoff and Cypionka, 2017). Untreated EA suspensions served as the negative control, whereas cells treated with 10% sodium hypochlorite functioned as the positive control. The identical experimental setup was applied to Xcc. All data were derived from three independent experiments, each performed in triplicate.

#### 2.3.5 Amylovoran production test

Amylovoran represents a major exopolysaccharide and key virulence determinant in *Erwinia amylovora* (EA), whose pathogenicity is primarily mediated by the type III secretion system (T3SS) and amylovoran biosynthesis. Quantification of amylovoran production was performed using the cetylpyridinium chloride (CPC) assay, as previously described by (Bellemann et al. (1994). Briefly, EA cultures were centrifuged overnight at 4°C, and the resulting cell pellets were washed with phosphate-buffered saline (PBS). The pellets were then diluted 1:100 into a modified Burkholderia minimal agar (MBMA) medium, composed of 3 g KH₂PO₄, 7 g K₂HPO₄, 1 g (NH₄)₂SO₄, 2 mL glycerol, 0.5 g citric acid, and 0.03 g MgSO₄, and further supplemented with 1% sorbitol and hop extracts at subMIC concentrations. Following incubation, the culture supernatants were collected and reacted with CPC by adding 50 μL of a 50,000 μg/mL CPC solution per mL of supernatant and incubating for 10 min at room temperature. Untreated EA cells served as the control. Amylovoran production was quantified by measuring turbidity at OD₆₀₀ using a spectrophotometer, and the results were interpreted based on a standard calibration curve correlating absorbance with amylovoran concentration. All experiments were conducted in triplicate across three independent biological replicates.

### 2.4 *In planta* and *ex planta* evaluations

#### 2.4.1 *In planta* activity of extracts on *Pyrus communis* trees

*Erwinia amylovora* (EA) strains were cultured in 5 mL of Luria-Bertani (LB) broth at 28°C for 24 h. Following incubation, bacterial cells were harvested by centrifugation and resuspended in phosphate-buffered saline (PBS) to obtain a final concentration of 10^7^ CFU/mL. Greenhouse-based pathogenicity assays were carried out on two-year-old *Pyrus communis* cv. “San Pietro” pear seedlings (Mendes et al., 2021). For each treatment group, three independent trees were used; on each tree, three separate shoots were selected for inoculation and subsequent treatment. Plants were maintained under controlled environmental conditions throughout the trial: 25°C ambient temperature, 70% relative humidity, and approximately 12 h of natural light per day. In the control group, a 50 μL aliquot of the EA suspension was introduced into actively growing shoots (15–20 cm in length) using scissor inoculation. For the treatment group, an identical inoculation protocol was followed, and after the appearance of disease symptoms (7 days post-inoculation), hop extracts at their minimum inhibitory concentration (MIC) were sprayed directly onto the infected shoots. Disease symptoms were monitored at 14 and 28 days post-inoculation. Both total shoot length and lesion (necrotic) length were measured, and the severity of fire blight infection was expressed as a disease index (DI), calculated as: DI = (lesion length/total shoot length) × 100. Each condition was evaluated in triplicate to ensure statistical robustness and reproducibility. Lesion areas were further quantified using ImageJ software (version 2.9, Fiji ImageJ for macOS, NIH, Maryland, United States), following the procedure described by Schneider et al. (2012).

#### 2.4.2 *Ex planta* activity of extracts on *Brassica* leaves

To evaluate the *ex-planta* antimicrobial efficacy of hop extracts, previously shown to possess bactericidal activity *in vitro*, against *Xanthomonas campestris* pv. *campestris* (Xcc) on *Brassica oleracea* var. *capitata* (cabbage), the following experimental protocol was employed. A total of 200 μL of Xcc bacterial suspension was inoculated into 5 mL of Luria-Bertani (LB) broth and incubated for 48 h at 28°C with agitation at 120 rpm. After incubation, cultures were centrifuged, and the bacterial pellet was resuspended in phosphate-buffered saline (PBS) to obtain a final concentration of 10^7^ CFU/mL. Four cabbage leaves of similar weight (variation within ±10%) were selected for analysis. Sterile scissors were immersed in the Xcc suspension, and three of the leaves were cut into uniform strips and placed into separate sterile plastic bags. The fourth leaf was similarly processed using sterile scissors without prior contact with the bacterial suspension, serving as the bacteria-free control. Two hydroalcoholic hop extracts (80:20 and 70:30 v/v ethanol:water) were tested at their minimum inhibitory concentration (MIC) of 1 mg/mL. Each extract was diluted in sterile water to a final volume of 5 mL and sprayed into its corresponding plastic bag. The remaining two bags, the negative control (uninfected leaf) and the positive control (infected, untreated leaf), received 5 mL of sterile water. All samples were sealed and stored at 4°C. From day 1 to day 30 of refrigerated storage, visual inspection was performed daily, and any necrotic lesions that developed on the cabbage leaves were documented and compared with control samples to assess antimicrobial efficacy.

### 2.5 Statistical analysis

Statistical analyses were conducted using GraphPad Prism Software v.9.0.0 for MacOS (GraphPad Software, San Diego, California, United States). Comparisons were made by using one-way and two-way ANOVA followed by Dunnett’s *post hoc* test to analyze the effect of extracts and their concentration on microbial growth and virulence factors. *p* ≤ 0.05 or *p* ≤ 0.01 was used depending on the analysis.

## 3 Results

### 3.1 Folin–Ciocalteu test and DPPH assay

Phenolic compounds are widely present in both edible and non-edible plants and are known for their various biological effects, particularly for their antioxidant properties. Plant materials rich in phenols are increasingly attracting interest in the food industry due to their ability to slow oxidative lipid degradation, thereby improving food quality and nutritional value. Moreover, these antioxidant compounds play a crucial role in health maintenance, contributing to the prevention of coronary heart disease and cancer (Agati et al., 2012; Kowalczyk et al., 2013).

To assess the total polyphenol content in the extracts, the Folin–Ciocalteu test was employed, and the results are summarized in Table 1.

**Table 1:** TPC in hop extracts by Folin–Ciocalteu test.

Extracts of HPW	TPC (μg GAE/mg)
Methanolic extract	48.4 ± 0.7
Ethanolic extract	44.9 ± 0.6
Hydroalcoholic extract (80:20)	58.3 ± 0.3
Hydroalcoholic extract (70:30)	47.7 ± 0.2

Standard deviation has been assessed by ANOVA (*p* = 0.1). TPC, total polyphenol content; HPW, hop waste powder. There was no statistically significant difference between extracts TPC.

From these values, it is evident that the total polyphenol content in the methanolic, ethanolic, and hydroalcoholic (70:30) extracts is comparable, while the hydroalcoholic extract (80:20) contains the highest amount of polyphenols. Notably, this extract also exhibited the highest extraction yield among all tested extracts. These findings align with literature reports; for instance, Gorjanović et al. found a total polyphenol content of 42.44 ± 0.88 μg GAE/mg in their methanolic extract, while Kowalczyk et al. (2013) and Gorjanović et al. (2013) analyzed hydroalcoholic extracts (50:50 methanol/water and ethanol/water) with values of 49.66 μg GAE/mg and 52.74 μg GAE/mg, respectively (Kowalczyk et al., 2013; Gorjanović et al., 2013). In facts, ethanol:water mixtures offer an optimal balance between polarity and solubility, enhancing the extraction of both polar and moderately non-polar phenolic compounds (Hrnčič et al., 2019).

**Figure 1:**
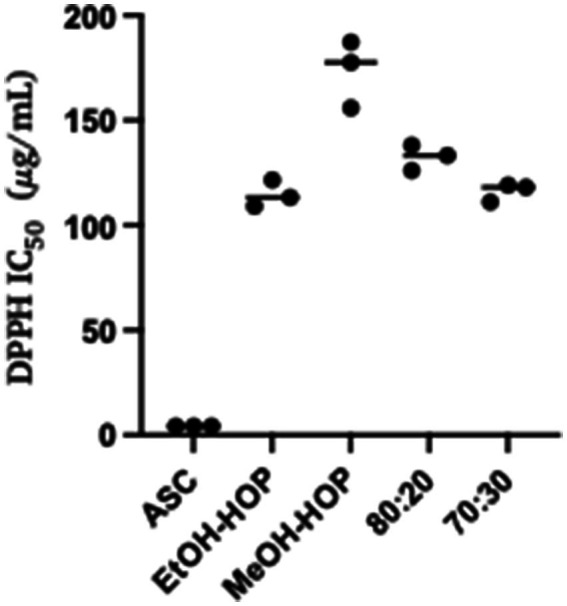
Antioxidant activity of hop waste powder extracts presented as DPPH IC_50_ compared to the control, ASC (ascorbic acid): EtOH-HOP, ethanol extract; MeOH-HOP, methanol extract; 80:20, hydroalcoholic extract 80:20; 70:30, hydroalcoholic extract 70:30.

The DPPH assay results reveal that the antioxidant activity of the compounds present in the ethanolic and hydroalcoholic extracts (70:30 and 80:20) is notably comparable (Figure 1), while the methanolic extract exhibits the lowest antioxidant capacity among all tested extracts. This variation in antioxidant activity can be attributed to the differences in the chemical composition and solubility of the bioactive compounds extracted by each solvent. Ethanol and hydroalcoholic mixtures are particularly effective at solubilizing a wide range of phenolic compounds and flavonoids, which are known for their strong antioxidant properties. These compounds can scavenge free radicals, thereby mitigating oxidative stress. In contrast, methanol may not extract the same diversity or concentration of these beneficial molecules, leading to its reduced efficacy in antioxidant activity (Azieana et al., 2017; Sreelatha and Padma, 2009). This finding suggests that the choice of solvent plays a critical role in maximizing the extraction of antioxidant compounds from HWP, highlighting the potential of ethanolic and hydroalcoholic extracts for applications in health and food preservation.

### 3.2 Minimal inhibitory and bactericidal concentration

The lowest concentration exhibiting a bacteriostatic effect was identified in the first well which showed no- or lower turbidity compared to the control. This result was confirmed by measuring absorbance using a microplate reader. For *EA*, wells with concentrations below 1 mg/mL displayed significant turbidity for all tested extracts, indicating that concentrations of 0.1 mg/mL and 0.01 mg/mL are insufficient to inhibit bacterial growth. In contrast, wells containing extracts at concentrations of 1 mg/mL and 10 mg/mL showed no turbidity, demonstrating bacteriostatic activity at these levels. Thus, we can define the minimum inhibitory concentration (MIC) for all extracts as 1 mg/mL. Solvent-only treatments were included as negative controls to isolate the effect of hop-derived compounds from the intrinsic antimicrobial activity of the solvents themselves. No effects were observed in solvent-only samples.

Regarding the minimum bactericidal concentration (MBC), we plated the 10 mg/mL and 1 mg/mL suspensions on LB agar plates to count colonies and confirm bactericidal action. The results indicated that the two alcohol extracts (methanol and ethanol) exhibited bactericidal activity against EA at a concentration of 10 mg/mL, while the two hydroalcoholic extracts did not show any bactericidal effects at this concentration, only bacteriostatic activity on EA.

For Xcc, wells below the 1 mg/mL concentration again showed significant turbidity for all tested extracts, confirming that concentrations of 0.1 mg/mL and 0.01 mg/mL are too low to affect bacterial growth. Conversely, wells with extracts at 1 mg/mL and 10 mg/mL exhibited no turbidity, indicating bacteriostatic activity at these concentrations. Therefore, the MIC against Xcc for all tested extracts is also 1 mg/mL.

For the MBC, we plated the wells containing suspensions at 10 mg/mL and 1 mg/mL on R2A agar plates to assess bacterial colonies and confirm bactericidal action. The results showed that the hydroalcoholic extracts (80:20 and 70:30) had bactericidal activity at 10 mg/mL, as no bacterial growth was observed. The ethanolic extract yielded a single colony, while the methanolic extract produced numerous colonies that were uncountable, although with lower growth than the control. Thus, we conclude that the methanolic extract does not exhibit bactericidal activity at 10 mg/mL but is bacteriostatic on Xcc.

### 3.3 Biofilm studies: biofilm formation and biofilm removal activity of the extracts

**Figure 2:**
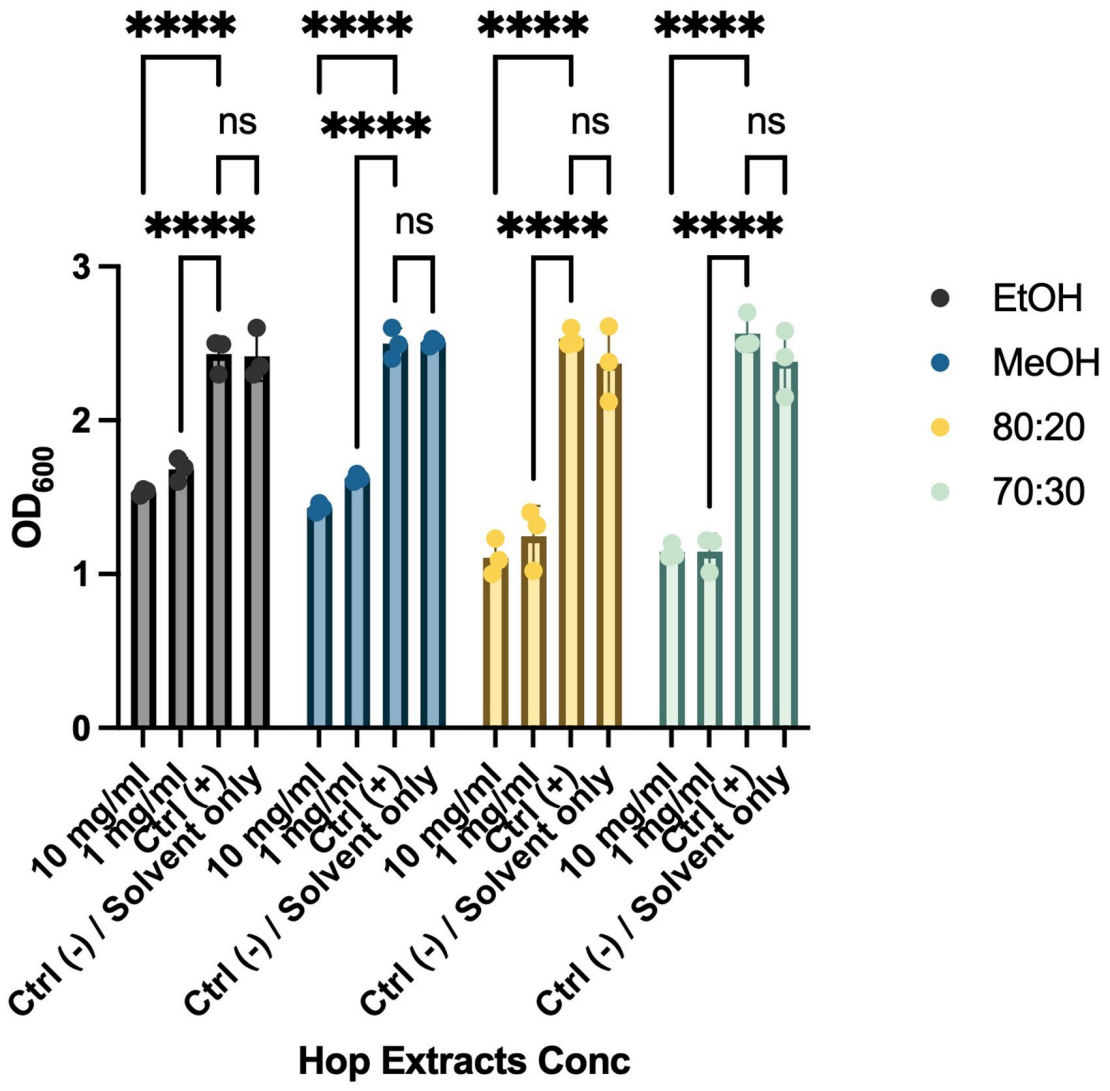
Effects of HWP at different concentrations on Biofilm formation processes of Xcc. Effects of hop waste powder extracts on Xcc biofilm formation are presented as absorbances (OD_600_) of crystal violet. EtOH, ethanol extract; MeOH, methanol extract; 80:20, hydroalcoholic extract 80:20; 70:30, hydroalcoholic extract 70:30; Ctrl+, positive control, untreated bacteria. **** *p* < 0.0001.

**Figure 3:**
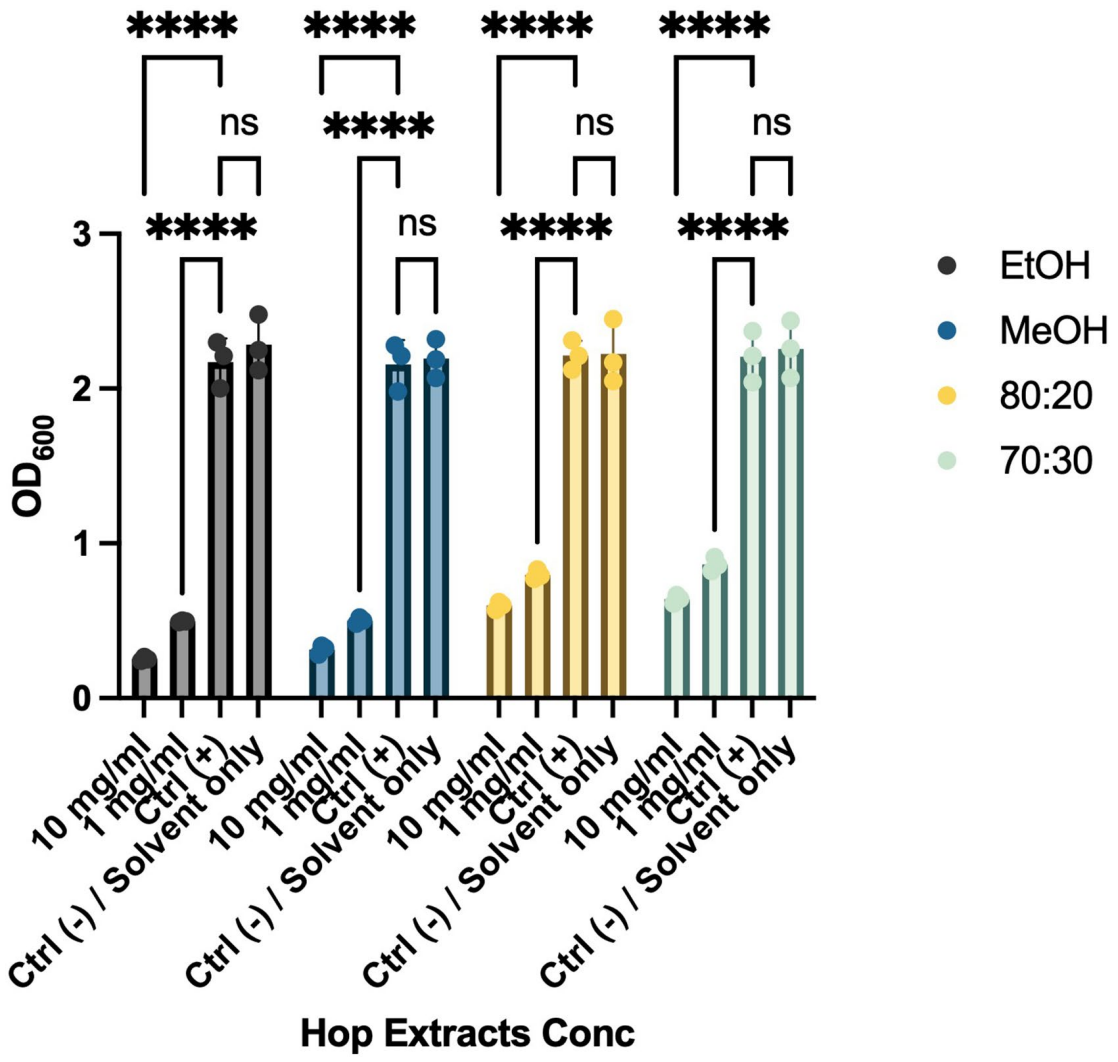
Effects of HWP at different concentrations on biofilm formation processes of EA. Effects of hop waste powder on EA biofilm formation are presented as absorbances (OD_600_) of crystal violet. EtOH, ethanol extract; MeOH, methanol extract; 80:20, hydroalcoholic extract 80:20; 70:30, hydroalcoholic extract 70:30; Ctrl+, positive control, untreated bacteria; **** *p* < 0.0001.

Biofilm formation is a critical mechanism in the pathogenesis of cruciferous black rot, caused by the Xcc bacterium, and fire blight, caused by EA. This phenomenon enables bacterial colonies to resist environmental stresses cohesively through various intercellular communication mechanisms. To combat bacterial biofilms, an effective strategy is to target different stages of biofilm development, including adhesion, motility, production of extracellular polymeric substances (EPS), and quorum sensing (QS) (Høiby et al., 2011; Sharma et al., 2023). These interventions can weaken, disperse, or disrupt biofilms, facilitating their removal. To evaluate the anti-biofilm activity of HWP extracts, we added bacteriostatic and/or bactericidal concentrations of hop powder (10 mg/mL and 1 mg/mL) to Xcc and EA bacterial suspensions. After incubation, we quantified biofilm formation using a spectrophotometer (Figures 2, 3). As shown in Figure 2, there was a significant decrease in biofilm formation for all tested extracts compared to the positive control, which consisted of the bacterial suspension alone. To exclude any potential effects of the solvents used in extract preparation, solvent-only (negative control) samples were included in the biofilm formation assay (Figures 2, 3). The results indicate that there were no statistically significant differences between the untreated control and the solvent-only treatments, confirming that the observed inhibition of biofilm formation was due to the bioactive compounds in the hop waste extracts rather than the solvents themselves.

**Figure 4:**
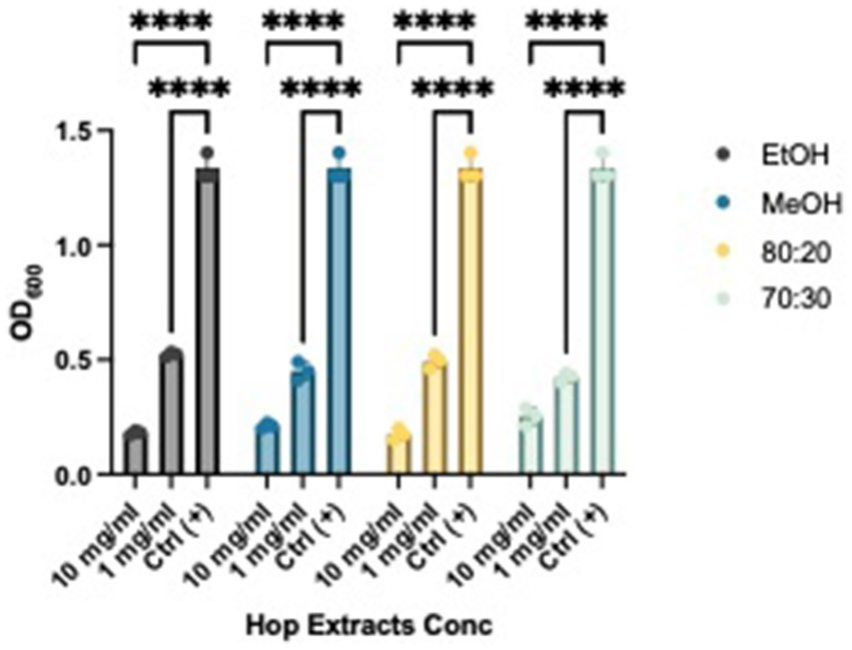
Effects of HWP at different concentrations on Xcc’s biofilm removal. Effects of hop waste powder extracts on Xcc biofilm formation are presented as Absorbances (OD_600_) of crystal violet; EtOH, ethanol extract; MeOH, methanol extract; 80:20, hydroalcoholic extract 80:20; 70:30, hydroalcoholic extract 70:30; Ctrl+, positive control, untreated bacteria; **** *p* < 0.0001.

**Figure 5:**
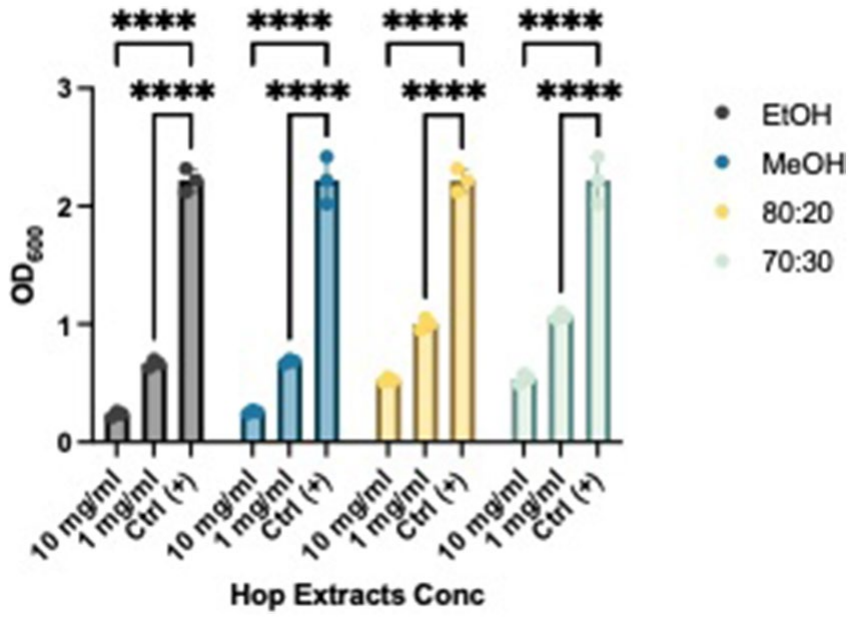
Effects of HWP at different concentrations on EA’s biofilm removal. Effects of hop waste powder extracts on Xcc biofilm formation are presented as absorbances (OD_600_) of crystal violet; EtOH, ethanol extract; MeOH, methanol extract; 80:20, hydroalcoholic extract 80:20; 70:30, hydroalcoholic extract 70:30; Ctrl+, positive control, untreated bacteria; **** *p* < 0.0001.

After establishing the anti-biofilm formation activity, we investigated whether the extracts could weaken and remove pre-formed biofilms. As shown in Figures 4 and 5, all extracts demonstrated the ability to remove biofilms of Xcc and EA, with a more pronounced effect at a concentration of 10 mg/mL compared to the control. Notably, they also exhibited activity at a concentration of 1 mg/mL. This evidence aligns with literature indicating that extracts from exhausted hop cones—free of xanthohumol but rich in various derivatives of quercetin, kaempferol, catechin, and epicatechin—along with pure xanthohumol, significantly reduced the viability of mature biofilms on *Staphylococcus aureus* when applied at relatively low concentrations (MIC or 2xMIC) (Panda et al., 2020; Rozalski et al., 2013).

### 3.4 Membrane permeability

**Figure 6:**
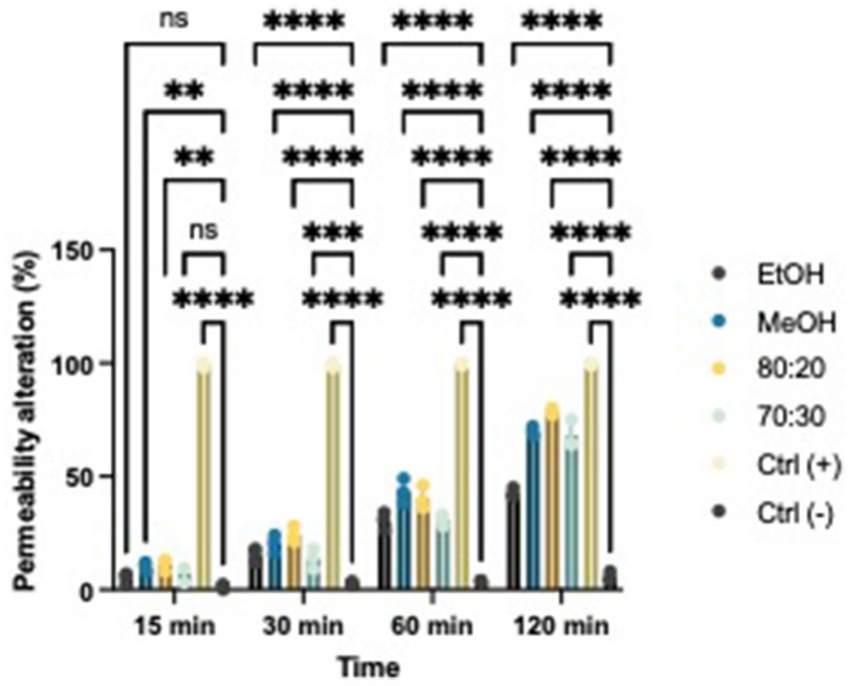
Effects of hop waste powder used at MIC/2 concentration on EA membrane permeability at *t* = 15 min, 30 min, 60 min, and 120 min. Permeability alteration is presented as a percentage. EtOH, ethanol extract; MeOH-HOP, methanol extract; 80:20, hydroalcoholic extract 80:20; 70:30, hydroalcoholic extract 70:30; Ctrl+, positive control, bacteria treated with bleach solution; Ctrl−, untreated bacteria; (ns), not significant, *p < 0.05, **p < 0.005, ***p < 0.0005, and ****p < 0.0001.

**Figure 7:**
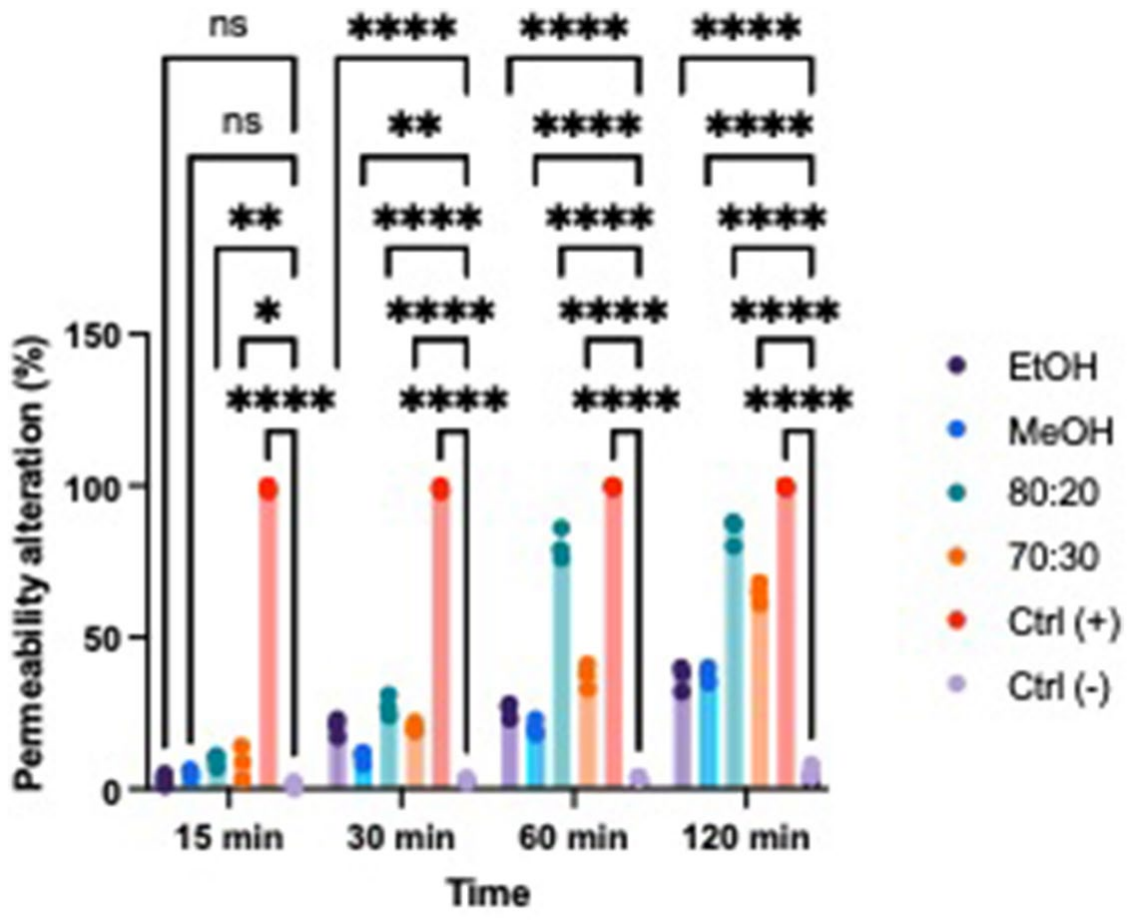
Effects of hop waste powder used at MIC/2 concentration on Xcc membrane permeability at *t* = 15 min, 30 min, 60 min, and 120 min. Permeability alteration is presented as a percentage. EtOH, ethanol extract; MeOH-HOP, methanol extract; 80:20, hydroalcoholic extract 80:20; 70:30, hydroalcoholic extract 70:30; Ctrl+, positive control, bacteria treated with bleach solution; Ctrl−, untreated bacteria; (ns), not significant, *p < 0.05, **p < 0.005, ***p < 0.0005, and ****p < 0.0001.

Based on the results, we evaluated whether extracts with proven antibacterial activity could affect the permeability of the bacterial membrane in both Xcc and EA. We tested two concentrations that exhibited bacteriostatic and/or bactericidal activity on standardized bacterial suspensions. To assess potential alterations in membrane integrity, we added a fluorescent intercalating agent, propidium iodide (PI), to the bacterial suspension in each well. Under normal conditions, PI cannot penetrate the membrane; however, if the membrane’s integrity is compromised, the dye enters the bacterial cell and intercalates between DNA bases. This binding increases the quantum yield by 20–30 times, allowing to differentiate between cells with intact membranes and those with damaged structures using a spectrofluorometer (Figures 6, 7) (Crowley et al., 2016; Kirchhoff and Cypionka, 2017). All extracts demonstrated the ability to alter the bacterial membrane, but the hydroalcoholic extract (80:20) was particularly notable, showing significant activity at the MIC concentration of 1 mg/mL for both Xcc and EA, comparable to the positive control. This extract also revealed the highest total polyphenol content in the Folin–Ciocalteu test, supporting findings in the literature that phenolic compounds primarily exert their antibacterial effects by damaging the bacterial membrane, altering permeability, polarization, and efflux pump activity (Khan et al., 2017; Pei et al., 2022; Hossain et al., 2021). Solvent-only treatments were included as negative controls to isolate the effect of hop-derived compounds from the intrinsic antimicrobial activity of the solvents themselves. Results confirm that the observed bioactivity was due to hop extract compounds rather than solvents.

### 3.5 Amylovoran production

**Figure 8:**
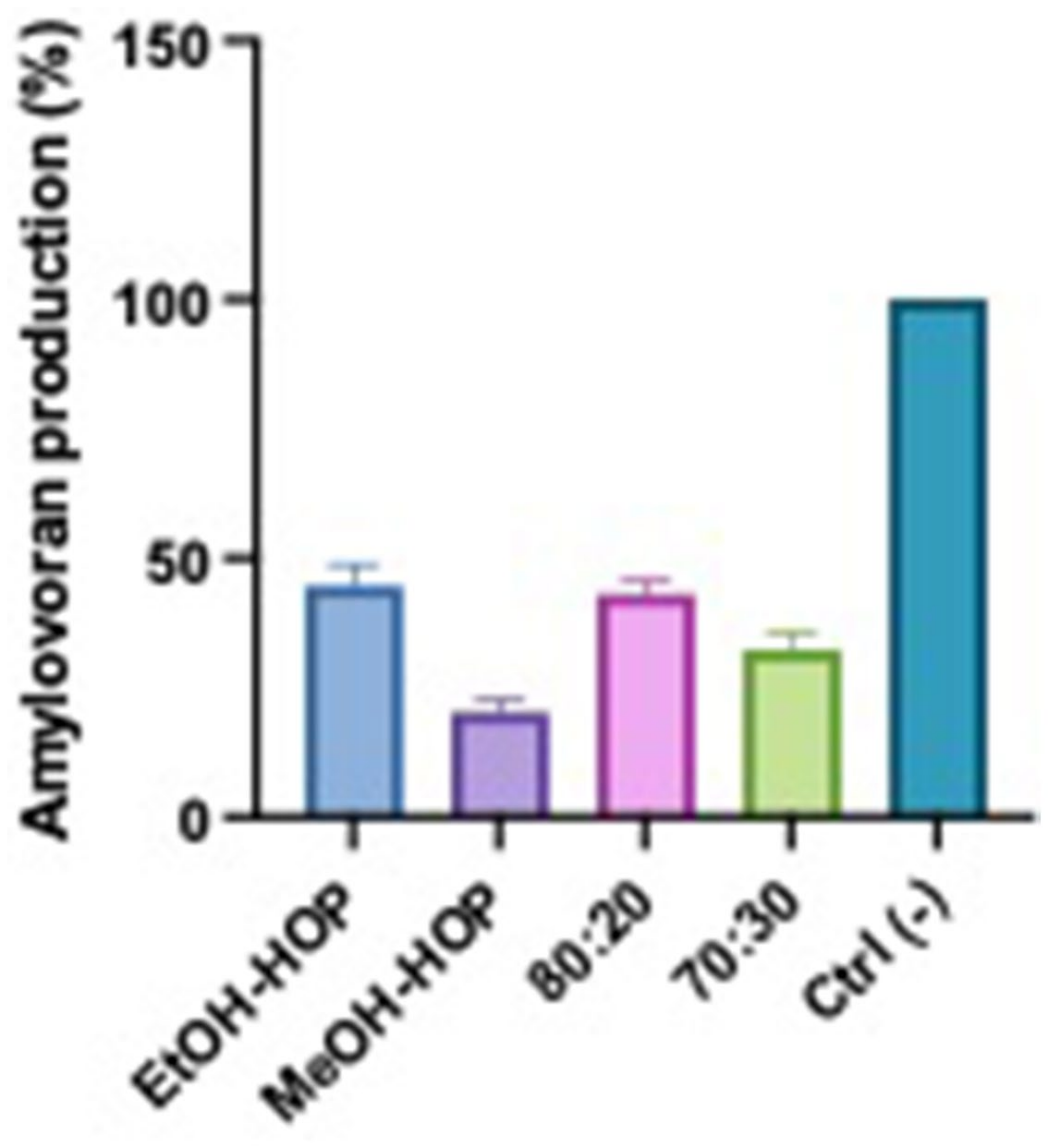
Effects of hop waste powder extracts on amylovoran production by EA. Amylovoran production is presented as a percentage compared to the negative control (Ctrl−), which consisted in untreated EA. EtOH-HOP, ethanol extract; MeOH-HOP, methanol extract; 80:20, hydroalcoholic extract 80:20; 70:30, hydroalcoholic extract 70:30.

The results of the amylovoran production assay indicate that hop waste powder extracts, especially those based on ethanol, significantly inhibit the production of this virulence factor in *Erwinia* species associated with fire blight. As seen in Figure 8, the higher inhibition (40–45%) observed with ethanolic extracts compared to the methanolic extracts (18%) may be attributed to the differences in solubility and extraction efficiency of bioactive compounds in these solvents (Hrnčič et al., 2019; Laws, 1981; Arráez-Román et al., 2006). Ethanol is known to effectively extract a broader range of phenolic compounds and flavonoids from hops, which possess antimicrobial and anti-virulence properties. These compounds may disrupt the metabolic pathways involved in amylovoran synthesis or interfere with the signaling mechanisms that regulate virulence factor production. In contrast, methanol, while also a good solvent, may not extract the same spectrum of active compounds, leading to a lesser inhibitory effect. This suggests that the specific phytochemicals present in the ethanolic extracts play a crucial role in mitigating the pathogenicity of *Erwinia*, highlighting the potential of hop extracts as a natural means to manage fire blight.

### 3.6 Analysis of *ex planta* antimicrobial effects on cabbage leaves

To evaluate the bactericidal capacity of the 80:20 and 70:30 hydroalcoholic extracts against the bacterium Xcc, we conducted a preliminary study to assess their antibacterial effects in a plant system. This experiment provides valuable insights into how Xcc, the causative agent of black rot in cruciferous plants, interacts with plant cells and how hop waste powder extracts perform in a more realistic three-dimensional context. In response to microbial attacks, infected plants activate various defensive mechanisms, both innate and acquired, which operate at different levels. Solvent-only treatments were included as negative controls to isolate the effect of hop-derived compounds from the intrinsic antimicrobial activity of the solvents themselves. No effects were observed in solvent-only samples.

**Figure 9:**
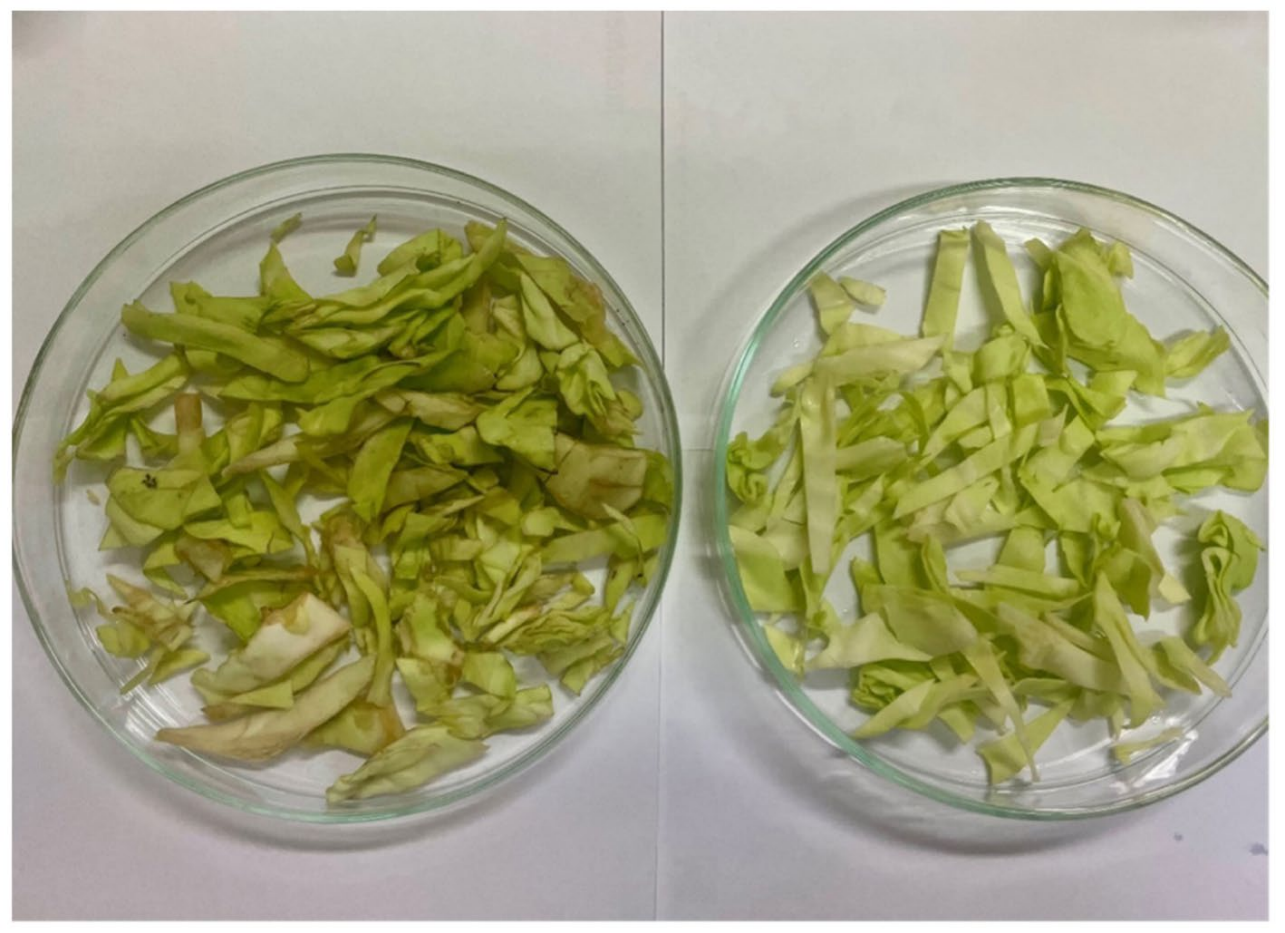
Day 8. Comparison of the two controls tested: on the left leaf infected with Xcc and untreated, which serves as a control, on the right leaf free of pathogen and not treated.

**Figure 10:**
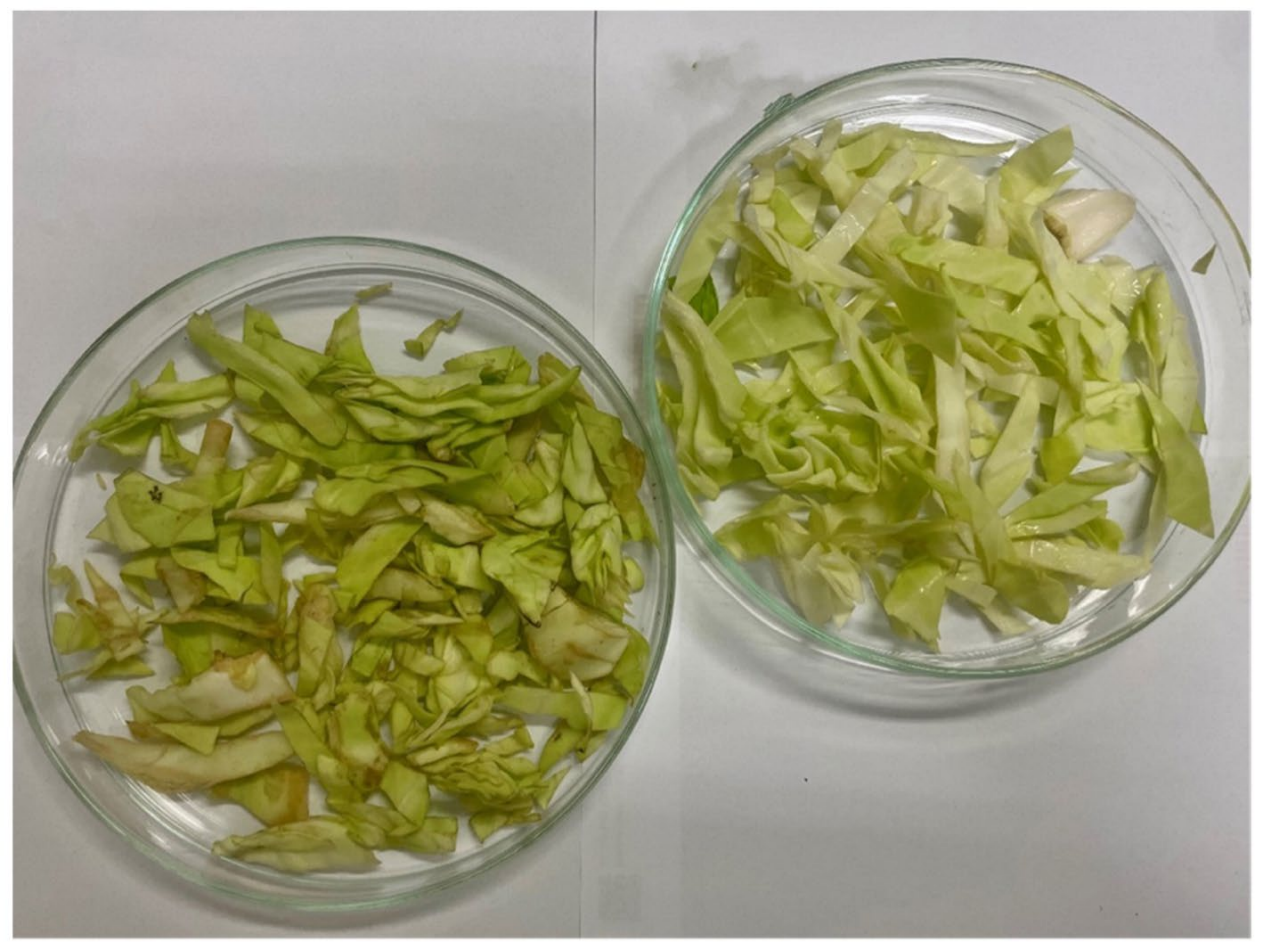
Day 8. On the left control infected with Xcc and untreated; on the right, leaf infected and treated with hydroalcoholic extract 80:20.

**Figure 11:**
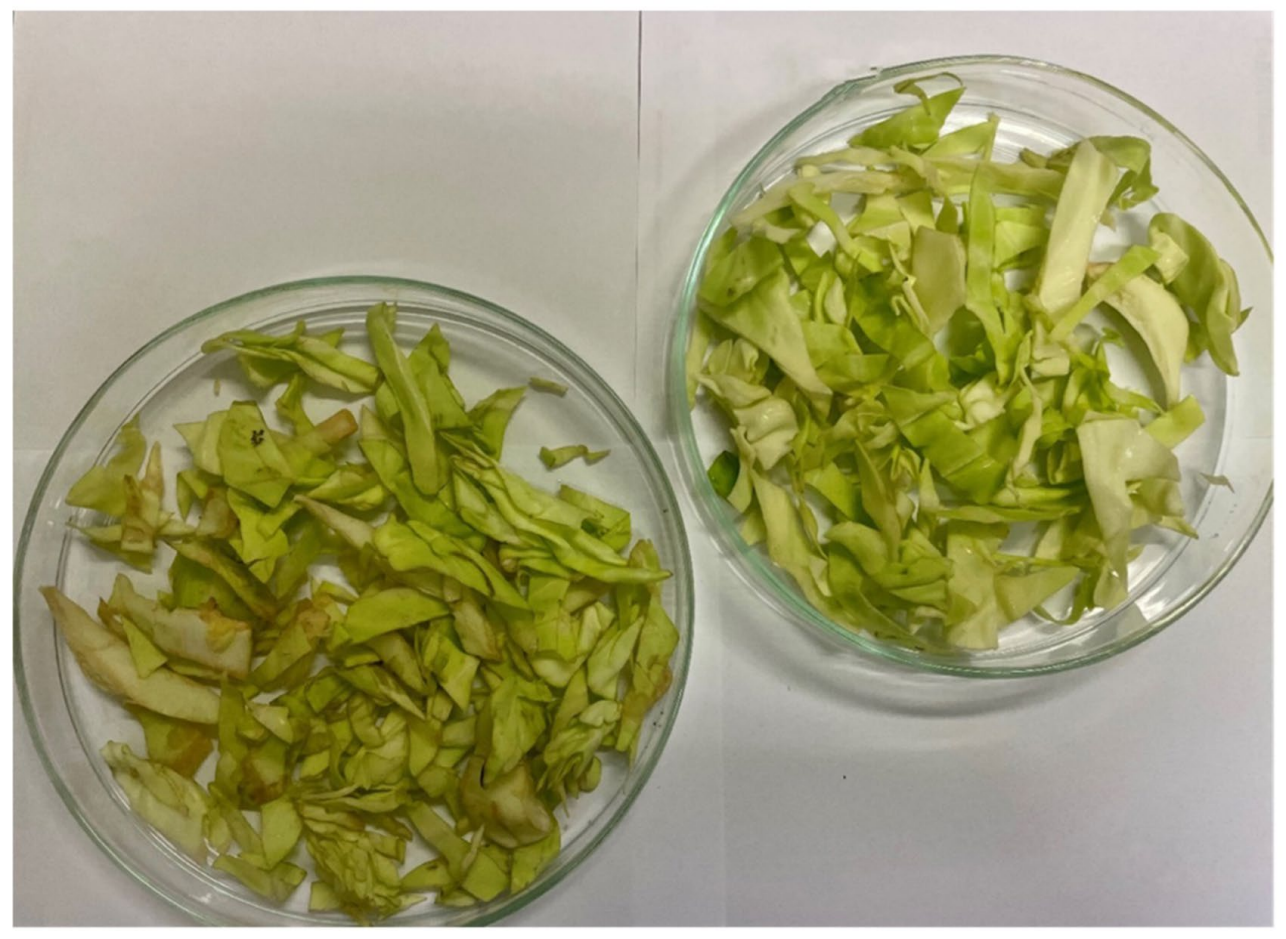
Day 8. On the left control infected with Xcc and untreated; on the right, leaf infected and treated with hydroalcoholic extract 70:30.

We tested the antimicrobial properties of these extracts on cabbage leaves infected with Xcc. After washing the leaf surfaces, we cut the leaves using scissors immersed in the bacterial suspension, simulating a natural infection mechanism. The cut leaves were then placed in small strips inside plastic bags, and the extracts were sprayed at their MIC concentrations. We stored the leaves at 4°C for 8 days, allowing sufficient time for the disease to develop. At the end of the incubation period, the leaves were transferred to glass Petri dishes for analysis of the infection area. The results obtained are illustrated in Figures 9, 10, and 11.

**Figure 12:**
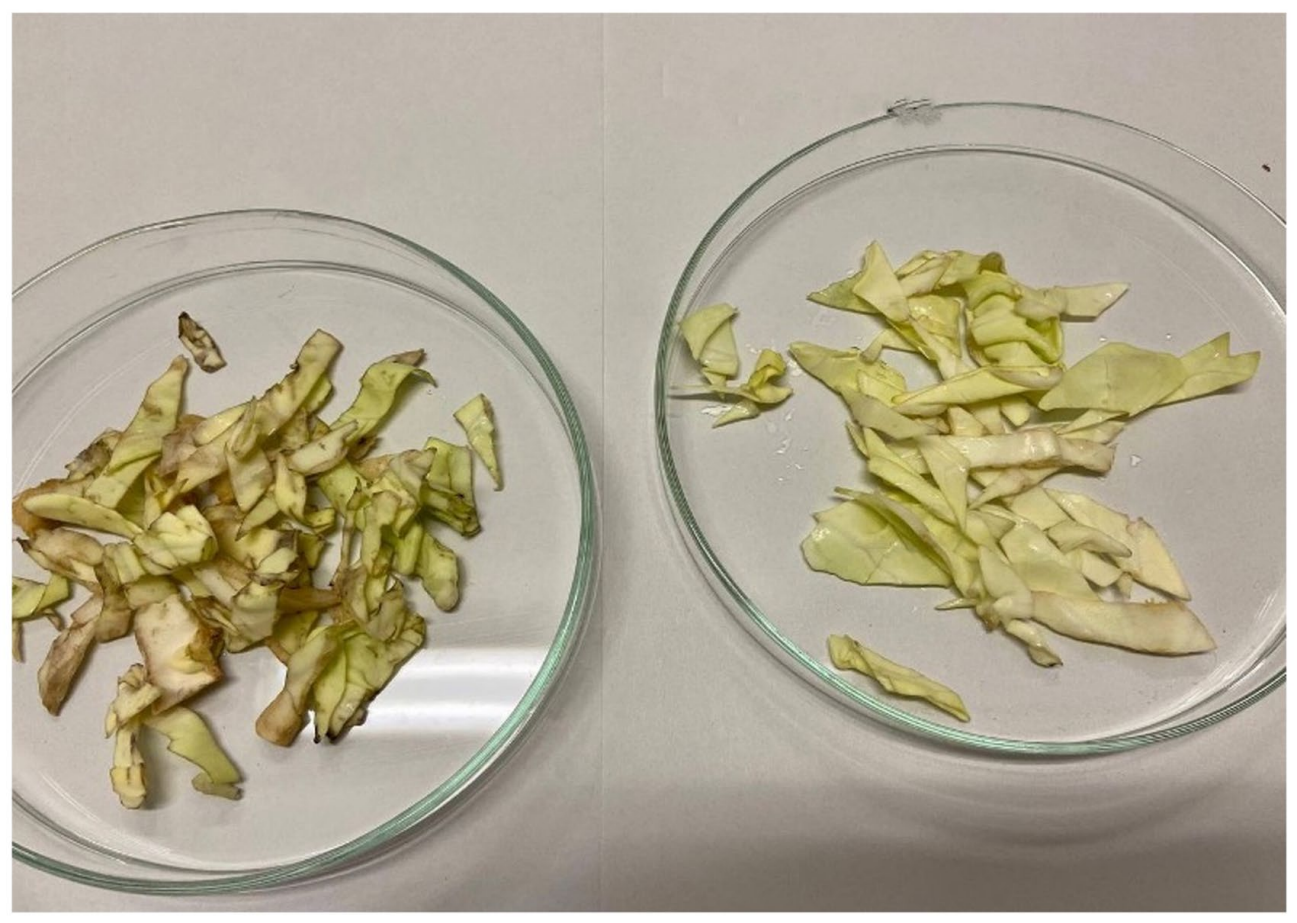
Day 30. On the left control infected with Xcc and untreated; on the right, leaf infected and treated with hydroalcoholic extract 80:20.

**Figure 13:**
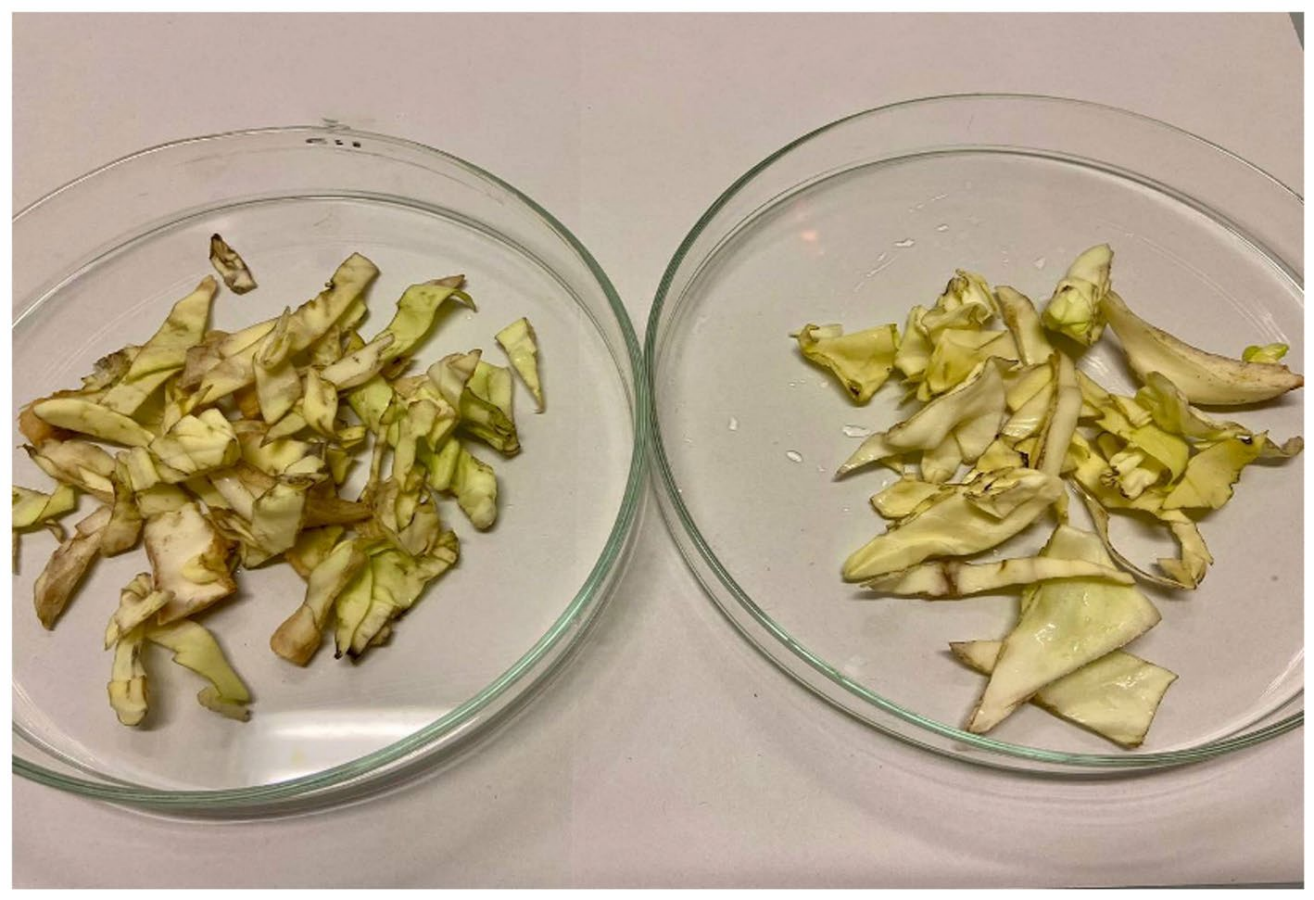
Day 8. On the left control leaves infected with Xcc and untreated; on the right, leaves infected and treated with hydroalcoholic extract 70:30.

As shown in the images, both tested extracts effectively block the infection caused by Xcc. Unlike the control group, which exhibits visible signs of rot, the leaves treated with the extracts show no signs of decay and remain comparable to day 1. As illustrated in Figures 12 and 13, even at the end of the experiment, after 30 days, the effects of the extracts are highly satisfactory. The control leaves have deteriorated further due to the progression of the bacterial infection, displaying rotten and blackened areas on their surfaces. In contrast, both extracts, particularly the hydroalcoholic 80:20, maintain leaf conditions similar to those observed on day 8. This demonstrates that the infection was effectively halted, ensuring the preservation of the leaves and protecting them from physiological deterioration.

The result obtained is not surprising, in fact in the literature several plant extracts (grape extracts, grapefruit seed extracts, and pomegranate extracts are just some examples) are reported as preservatives in fruit and vegetables due to the presence of polyphenols which have antimicrobial activities (Quideau et al., 2011; Ribeiro et al., 2016; Andrade et al., 2019; Shahbaz et al., 2022).

After establishing the *in vivo* activity of the hydroalcoholic HWP extracts, we conducted further analysis to determine the presence of Xcc in the plant structures. Following treatment of the cabbage leaves with the hop extracts, we assessed whether the bacterium was still present. The number of colony-forming units (CFU) detected in each analysis is reported in Table 2.

**Table 2:** Total viable count of Xcc colonies on LB agar after hop treatment of cabbage leaves.

Samples	CFU/mL
Leaf treated with hydroalcoholic extract (80:20)	45 ± 18
Leaf treated with hydroalcoholic extract (70:30)	90 ± 22
Untreated leaves	70.000 ± 138

Standard deviation has been assessed by ANOVA (*p* = 0.1).

The colonies from the control group, represented by the infected and untreated leaves, demonstrate high bacterial activity. In contrast, the treated leaves showed a significantly lower number of colonies, not exceeding 100 units. Notably, the hydroalcoholic extract (80:20) was the most active and effective. Overall, we can conclude that the pathogenesis of Xcc has significantly decreased, as the treated cabbage portions appeared visually less affected. Thus, Xcc persists in low quantities in the leaves and is not pathogenic.

### 3.7 Analysis of *in planta* antimicrobial effect in *Pyrus communis* plants

The potential of ethanolic and methanolic extracts from hops powder to combat EA *in vitro* was assessed, followed by a preliminary study to investigate their antibacterial effects *in planta*. This experiment provides insights into how the bacterium interacts with plant cells and the effects of the extracts in a more realistic three-dimensional context (Mendes et al., 2021; Gusberti et al., 2015).

**Figure 14:**
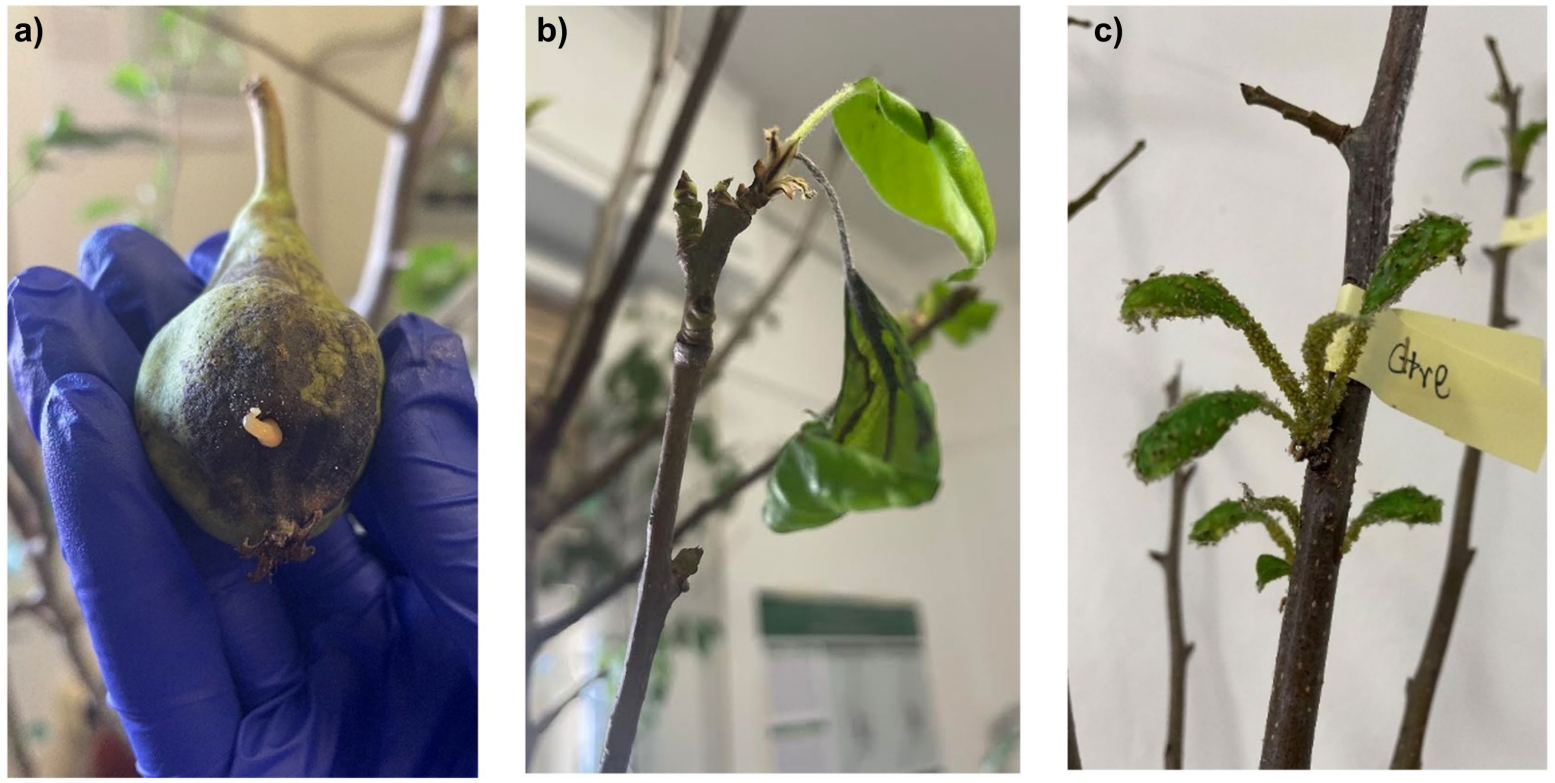
**(a)** Negative control, 7-day post-inoculation pear with bacterial “ooze” exudate; **(b)** negative control, 25-day post-inoculation leaf hook folding; **(c)** 25-day post-inoculation insect-rich negative control.

**Figure 15:**
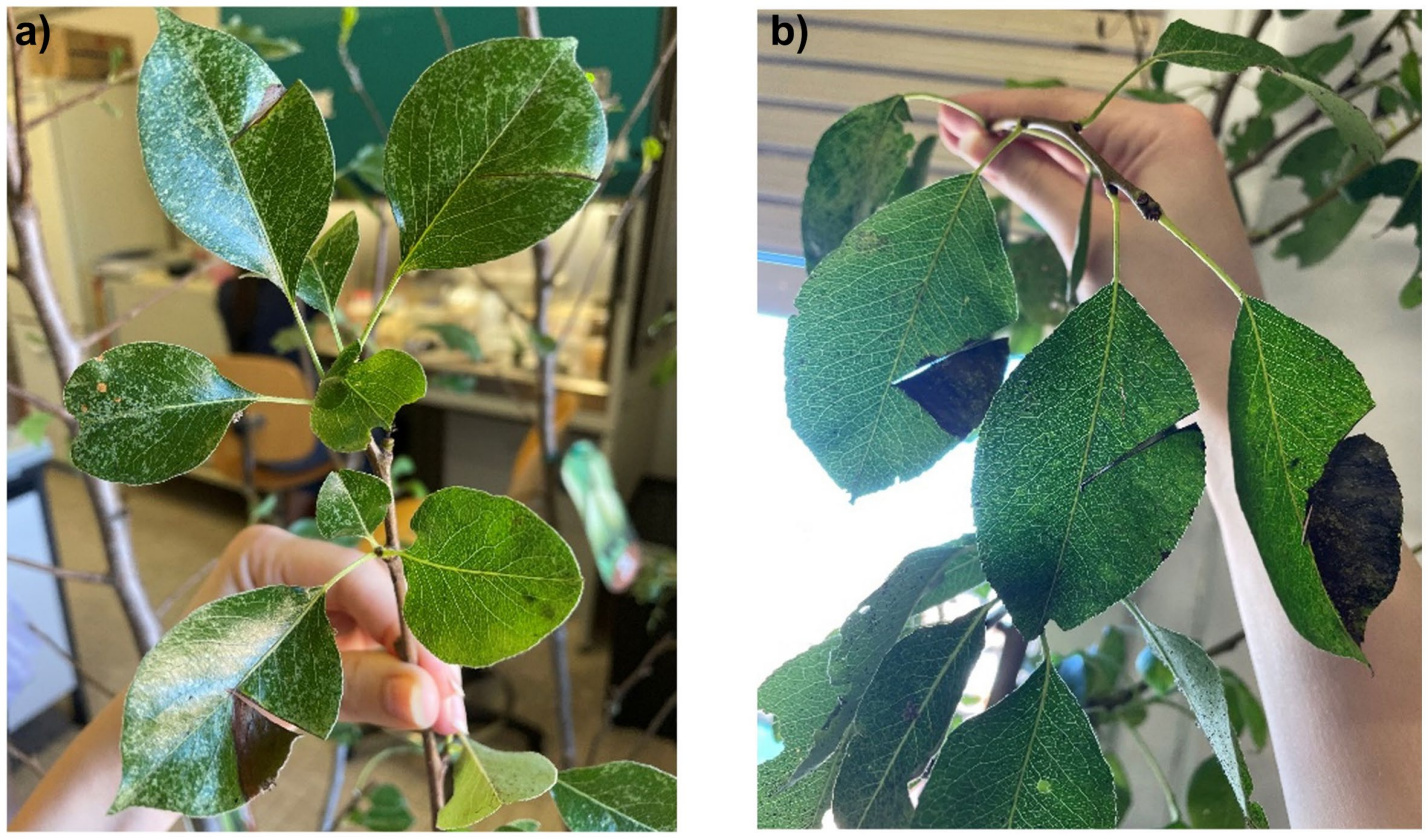
**(a)** infected pear pretreated with methanolic extract (sx) and negative control at 15 days post-inoculation; **(b)** comparison between negative control (sx) and plant treated with the extracts of hop waste powder (dx) at 25 days post-inoculation.

As EA is the causative agent of fire blight in *Rosaceae*, particularly in pears and apple trees, we tested the antimicrobial properties of these extracts on previously infected pear plants. As shown in Figures 14, 15b, the negative control group exhibited significant leaf loss, with the remaining leaves displaying the characteristic hook-like folding associated with fire blight. Additionally, after 7 days, bacterial exudate was observed on the surface of the infected pears, and the branches were infested with insects, unlike the treated plants, suggesting a possible insecticidal action of the extracts.

**Figure 16:**
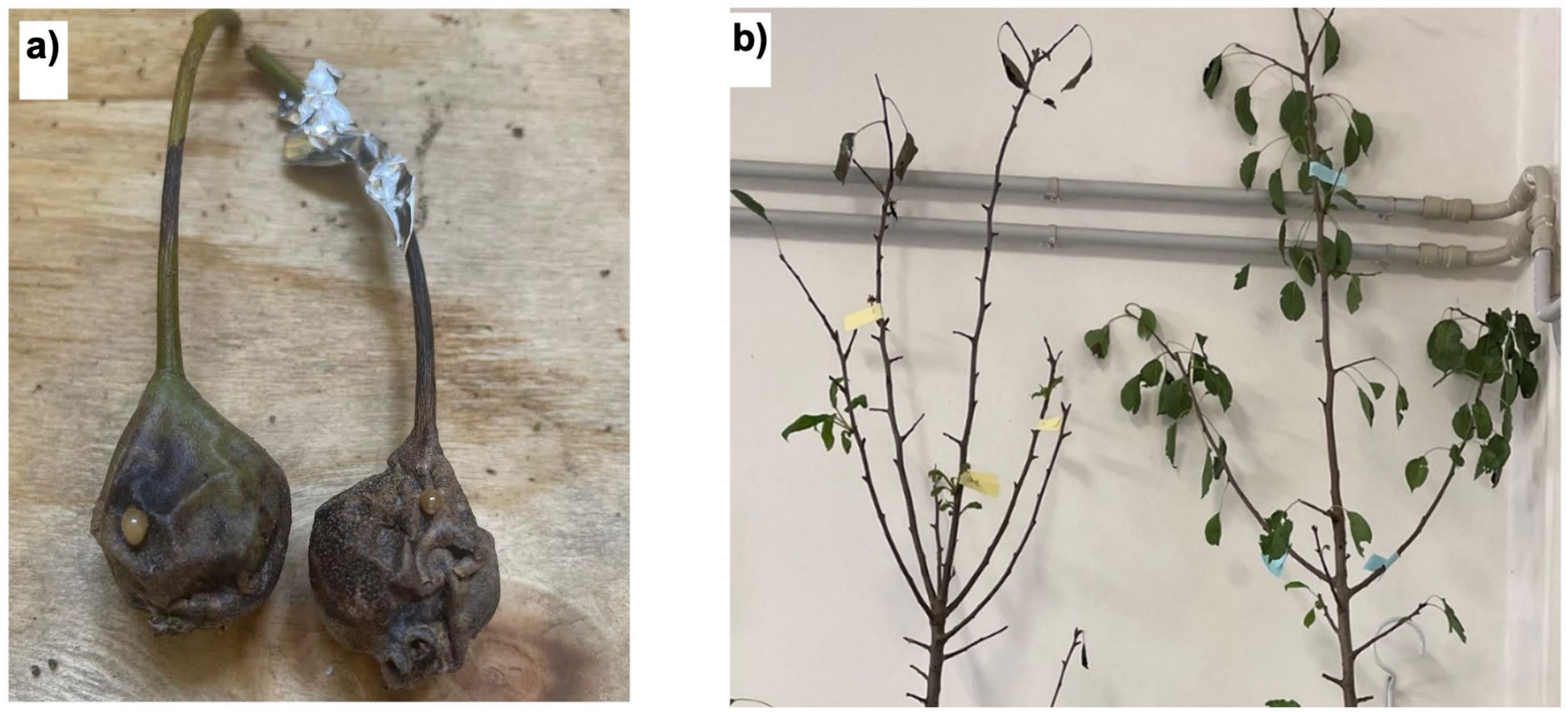
**(a)** Infected branch treated with methanolic extract of hop waste powder at 25 days post-inoculation; **(b)** infected branch treated with ethanol extract of hop waste powder at 25 days post-inoculation.

Analyzing the therapeutic effects, Figure 16 illustrates that both extracts effectively maintained the infected area, which remained largely unaltered after 25 days. In contrast to the control, the leaves on treated plants remained attached to the branches, with limited necrotic areas. Thus, the alcoholic extracts of HWP demonstrate promising therapeutic potential for treating fire blight.

From the results, it appears that the methanolic extract of HWP is more effective, particularly when applied preventively. However, as shown in Figure 15a, the preventive treatment on the pears was not successful, likely due to suboptimal administration or formulation of the extract. It is possible that after 2 days, the extract may have evaporated, failing to remain on the leaf surfaces. Therefore, a more effective and persistent formulation should be developed.

Spraying the extracts therapeutically is effective when the pear surfaces exhibit damage, allowing the extract to penetrate and act directly. There may also be a synergistic effect between the bioactive molecules in the extracts and the secondary metabolites already present in the plant. Studies by Shaw et al. indicate that the accumulation of phenolic compounds at the infection site strengthens the cell wall, accompanied by localized production of reactive oxygen species, which contributes to antimicrobial action (Shaw et al., 2006).

## 4 Discussion and conclusion

Black rot and fire blight diseases have a significant impact on agriculture, especially from an economic perspective, due to the lack of effective bactericides, the environmental impact of certain pesticides, and the development of resistance caused by the excessive use of non-specific antimicrobials. The need to reduce the use of toxic products for both the environment and human health, as well as the emergence of resistant bacteria, has driven research to explore more sustainable and organic alternatives. Among these alternatives, the use of phytocomplexes with antimicrobial properties has emerged as an effective and eco-friendly strategy.

This study aimed to analyze the antibacterial activity of phytocomplexes present in extracts obtained from drying and powdering whole hop biomass left after cone harvesting, commonly considered waste, against the phytopathogens *Xanthomonas campestris* pv. *campestris* (Xcc) and *Erwinia amylovora* (EA). The *in vitro* and *in planta* results confirm that hydroalcoholic and ethanolic extracts of HWP are rich in phenolic compounds and can inhibit bacterial growth and biofilm formation through multiple complementary mechanisms. A preliminary analysis demonstrated bacteriostatic activity at 1 mg/mL across all extracts, while hydroalcoholic extracts were bactericidal at 10 mg/mL against Xcc.

The antimicrobial effect appears to derive from the synergistic action of several polyphenolic compounds. These include prenylflavonoids and phenolic acids (e.g., naringenin, chlorogenic, ferulic, and ellagic acid), which interact with bacterial membranes, altering their permeability and fluidity, and impairing energy metabolism and nutrient uptake (Hossain et al., 2021; Mackieh et al., 2023; Lou et al., 2011). Other molecules such as epigallocatechin, quercetin, and kaempferol inhibit DNA/RNA synthesis and enzymes like DNA gyrase (Plaper et al., 2003; Periferakis et al., 2022). Additionally, *α*- and *β*-acids may dissipate the proton motive force by proton influx (Arruda et al., 2021; Schurr et al., 2015), while flavonols like rutin interfere with bacterial topoisomerases and penicillin-binding proteins (Amin et al., 2015; Ivanov et al., 2022; Alnour et al., 2022). Phenolic compounds may also irreversibly complex with membrane proteins or interfere with fatty acid and peptidoglycan biosynthesis (Scalbert, 1991; Brown et al., 2007; Bibens et al., 2023). These structural alterations may explain the membrane-disrupting effects observed, particularly with the 80:20 hydroalcoholic extract, which also exhibited the highest polyphenol content.

Beyond antibacterial action, several molecules, including catechins, tannins, quercetin, and ellagic acid, are known to interfere with biofilm formation, either by inhibiting cell adhesion or biofilm maturation (Ikigai et al., 1993; Stapleton et al., 2004; Wang et al., 2022; Hu and Pan, 2022; Buchmann et al., 2022). This is consistent with the observed inhibition and removal of EA and Xcc biofilms by HWP extracts, particularly at higher concentrations.

Importantly, this study extends beyond *in vitro* assays. The *ex planta* evaluation on cabbage leaves demonstrated that hydroalcoholic HWP extracts prevented visible decay and reduced viable bacterial counts over 30 days, highlighting their potential as a natural preservative. These results are in line with literature reports on the antimicrobial efficacy of polyphenol-rich plant extracts in food systems (Panzella et al., 2020; Conte et al., 2007; Burt, 2004; Tiwari et al., 2009; Deabes et al., 2021; Bulkan et al., 2022). Furthermore, the potential application in the food industry is underscored by the extracts’ low cost, minimal toxicity, and their dual role as antimicrobials and antioxidants.

An *in vivo* use of the tested extracts could not only combat plant infections but also support post-harvest preservation. In this regard, integration into natural food packaging materials or nanoformulations could enable targeted delivery and sustained antimicrobial activity. Recent advances in nanoencapsulation and polymer-based coatings offer promising avenues for such applications (Pettinato et al., 2017; Papoutsis et al., 2018).

Despite the strong bioactivity observed, the study has limitations. The persistence of the extracts on plant surfaces, their long-term stability, and solvent residue levels were not evaluated. Moreover, ethanol and methanol, while effective solvents, may be cost- or safety-limiting for large-scale use. Future studies should evaluate greener alternatives such as aqueous extractions, enzymatic digestion, or supercritical CO₂ extraction (Hrnčič et al., 2019; Panzella et al., 2020).

It is also essential to deepen our understanding of the specific compounds responsible for the observed effects. While total phenolics were quantified, future research should focus on identifying and quantifying key bioactives using targeted phytochemical techniques (e.g., HPLC, LC–MS, NMR). Furthermore, structure–activity relationship (SAR) analyses could help pinpoint the molecular determinants of efficacy.

From a translational perspective, scalability will depend on the reproducibility of extraction, regulatory approval, formulation development, and cost–benefit analyses. Encapsulation into biopolymers, controlled release systems, and compatibility with integrated pest management (IPM) strategies should be prioritized. Ultimately, extensive field trials will be necessary to confirm efficacy under real-world agronomic conditions.

In conclusion, hop biomass waste extracts, particularly hydroalcoholic formulations, represent a promising, sustainable, and circular economy-aligned approach for controlling plant bacterial diseases. Their effectiveness, affordability, and multi-target action suggest they could serve as valuable alternatives to synthetic pesticides and preservatives in both agricultural and food applications.

## Data Availability

The original contributions presented in the study are included in the article/supplementary material, further inquiries can be directed to the corresponding authors.
